# RNRdb, a curated database of the universal enzyme family ribonucleotide reductase, reveals a high level of misannotation in sequences deposited to Genbank

**DOI:** 10.1186/1471-2164-10-589

**Published:** 2009-12-08

**Authors:** Daniel Lundin, Eduard Torrents, Anthony M Poole, Britt-Marie Sjöberg

**Affiliations:** 1 Department of Molecular Biology and Functional Genomics, Stockholm University, Stockholm, Sweden; 2 Cellular Biotechnology, Institute for Bioengineering of Catalonia (IBEC), Scientific Park of Barcelona, Barcelona, Spain; 3 School of Biological Sciences, University of Canterbury, Christchurch, New Zealand

## Abstract

**Background:**

Ribonucleotide reductases (RNRs) catalyse the only known de novo pathway for deoxyribonucleotide synthesis, and are therefore essential to DNA-based life. While ribonucleotide reduction has a single evolutionary origin, significant differences between RNRs nevertheless exist, notably in cofactor requirements, subunit composition and allosteric regulation. These differences result in distinct operational constraints (anaerobicity, iron/oxygen dependence and cobalamin dependence), and form the basis for the classification of RNRs into three classes.

**Description:**

In RNRdb (Ribonucleotide Reductase database), we have collated and curated all known RNR protein sequences with the aim of providing a resource for exploration of RNR diversity and distribution. By comparing expert manual annotations with annotations stored in Genbank, we find that significant inaccuracies exist in larger databases. To our surprise, only 23% of protein sequences included in RNRdb are correctly annotated across the key attributes of class, role and function, with 17% being incorrectly annotated across all three categories. This illustrates the utility of specialist databases for applications where a high degree of annotation accuracy may be important. The database houses information on annotation, distribution and diversity of RNRs, and links to solved RNR structures, and can be searched through a BLAST interface. RNRdb is accessible through a public web interface at http://rnrdb.molbio.su.se.

**Conclusion:**

RNRdb is a specialist database that provides a reliable annotation and classification resource for RNR proteins, as well as a tool to explore distribution patterns of RNR classes. The recent expansion in available genome sequence data have provided us with a picture of RNR distribution that is more complex than believed only a few years ago; our database indicates that RNRs of all three classes are found across all three cellular domains. Moreover, we find a number of organisms that encode all three classes.

## Background

Ribonucleotide reductases (RNRs) form a universal enzyme family that catalyse the reduction of ribonucleotides to their corresponding deoxyribonucleotides. Ribonucleotide reduction provides the sole biological means for de novo synthesis of the building blocks of DNA, making it an essential cellular function. Sequence and structural data indicate that ribonucleotide reduction has evolved only once during evolution [1,2] and all RNRs make use of a common thiyl radical-based mechanism for catalysis [3].

In spite of the early evolutionary origins of ribonucleotide reduction, the modern diversity of RNRs is characterised by distinct biochemical routes to radical generation [4-6]. Three classes of RNR have been described on this basis, and each operates under a specific set of biochemical and environmental conditions (Table 1). Class I RNRs contain either a diiron centre that, via oxygen-mediated oxidation, generates a protein-based tyrosyl radical as a prerequisite to catalysis, or a Mn^IV^-Fe^III ^metal centre that substitutes for the tyrosyl radical. Consequently, class I RNR activity is dependent on the availability of molecular oxygen. Class II enzymes generate a radical via cleavage of vitamin B_12 _coenzyme (5'-deoxyadenosylcobalamin) and operate independent of oxygen. Class III enzymes also acquire a radical via cofactor cleavage (S-adenosylmethionine). Whereas class II RNRs must cleave and regenerate adenosylcobalamin with every round of catalysis, in class III RNRs the radical generated by cofactor cleavage (by action of an activase protein) is subsequently maintained as a stable protein-based glycyl radical. The glycyl radical of class III RNR is sensitive to oxygen; exposure results in enzyme destruction through backbone cleavage. Consequently, class III enzymes are inactivated even under microaerophilic conditions. For comprehensive reviews of the biochemistry of ribonucleotide reduction, see [3-6].

**Table 1:** General characteristics of RNR classes

	Class I	Class II	Class III
Operation	Aerobic	Oxygen independent	Anaerobic

Structure	α_2 _β_2_	α or α_2_	α_2_

Subunit names	Ia: NrdA, NrdB	NrdJ	NrdD
	Ib: NrdE, NrdF		specific activase: NrdG

Radical/cofactor	Tyr122 (in β)	AdoCbl	Gly580 (in α)

Reductant	Ia: Thioredoxin/Glutaredoxin	Thioredoxin	Formate
	Ib: NrdH-redoxin		

In archaea	Limited distribution	Yes	Yes

In bacteria	Yes	Yes	Yes

In eukaryotes	Yes	Limited distribution	Limited distribution

In viruses^a^	Yes	Bacteriophage; one eukaryotic virus	Bacteriophage

The catalytic subunits, NrdA, NrdE, NrdJ and NrdD are homologous, as are the two different class I radical generating subunits, NrdB and NrdF. The class III activase, NrdG, is not homologous to any other RNR subunit. Residue numbering refers to the *Escherichia coli *K12 class I radical generating subunit and the Bacteriophage T4 class III enzyme sequences respectively. Adapted from [2].

^a^RNR genes are only found in some dsDNA viruses.

Given that the propensity to synthesise deoxyribonucleotides is essential for DNA replication, the operational differences between classes of RNR suggest that the type of RNR any given organism carries will have an impact on the environmental conditions in which that organism can grow and reproduce. The effect on the biochemistry of ribonucleotide reduction of environmental parameters such as iron-, cobalt- and oxygen-availability may thus impact our understanding of the adaptability of microorganisms to a range of environments. An overview of the distribution and diversity of RNR classes -- particularly among microbes -- is therefore of interest in delimiting environmental range. To facilitate progress in these areas, we have established the Ribonucleotide Reductase database (RNRdb), a manually annotated and curated data source for annotation and comparative investigations centred on RNR biology.

## Construction and content of RNRdb

RNRdb is implemented using a relational database with an HTML user interface, and is available at http://rnrdb.molbio.su.se. The different proteins in RNRdb are denoted Nrd with appropriate suffixes (Tables 1, 2), according to the common nomenclature for the corresponding genes in bacteria and archaea; when applicable synonymous names are specified for each entry. NrdA and NrdB denote the components of the class Ia RNR, where NrdA contains the active site region and binding sites for allosteric effectors, and NrdB carries the stable tyrosyl radical. NrdB proteins with a Mn^IV^-Fe^III ^metal centre substituting the role of the tyrosyl radical [7,8] are denoted NrdB^Phe ^or NrdB^Leu^. The RNR components of class Ib are NrdE with the active site and allosteric binding regions and NrdF with the stable tyrosyl radical. In addition, class Ib operons code for NrdI, a flavoprotein, and often NrdH, a specific physiological reductant for the class Ib RNRs [9-12]. Class II RNRs are denoted NrdJ. The class III RNR proper is denoted NrdD. This RNR requires a specific activase, an iron-sulphur protein denoted NrdG [13] that belongs to the radical SAM protein family [14]. A majority of bacteria, and some archaea, encode the global regulator NrdR [15] that controls the transcription/translation of different RNR genes [16,17] (Table 2).

**Table 2:** Distribution of RNR proteins

Class	Subunit^a^	Archaea	Archaeal viruses	Bacteria	Bacteriophages	Eukaryotes	Eukaryotic viruses	Totals
Ia	NrdA	8	2	893	39	188	124	1254
	
	NrdB	8	2	910	47	247	140	1354

Ib	NrdE			605	7			612
	
	NrdF			647	8			655
	
	NrdH			393	7			400
	
	NrdI			633	5			638

II	NrdJ	62	4	702	18	7	1	794

III	NrdD	49		950	30	8		1037
	
	NrdG	48		923	21			992

All classes	NrdR	6		1268				1274

Totals		181	8	7924	182	450	265	9010

Distribution of RNR classes and NrdR regulator among cellular domains and viruses.

^a ^Protein roles for subunits are catalytic component (NrdA, NrdE), radical harbouring component (NrdB, NrdF), reductant (NrdH), flavodoxin component (NrdI), core enzyme (NrdJ, NrdD), activase (NrdG), and transcriptional regulator (NrdR).

**Figure 1:**
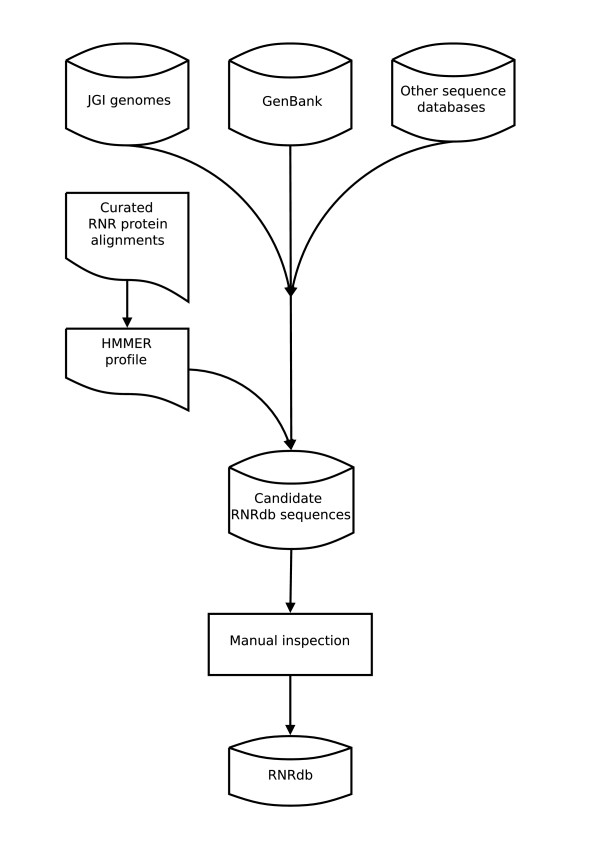
**RNRdb pipeline**. RNRdb is loaded from upstream databases (see text for details) using a semi automated pipeline. Before inclusion, each sequence is manually vetted.

The database was initially populated using manually collected and curated data, and is expanded and maintained as follows: Profile hidden markov models (HMM) [18] are generated, using HMMER [19], from alignments of known RNR sequences in the database, representing known sequence diversity for each RNR protein. Candidate protein sequences are then retrieved from GenBank using HMMER. Candidate sequences are filtered for duplications and manually checked before incorporation into RNRdb (Fig. 1). Manual curation is performed by aligning candidates to known experimentally annotated RNR sequences (a procedure for which there is theoretical precedent [20]), to ensure only full-length sequences possessing all key sequence motifs are inserted. NrdH and NrdG candidates pose special problems; NrdH because it is less than 100 residues and has striking similarities to thioredoxins/glutaredoxins [9,10], and NrdG because it is a member of the highly conserved radical SAM family [14] and often confused with pyruvate formate-lyase activating enzyme. For this reason an NrdH candidate is only accepted if located close to other class Ib members (NrdE, NrdF, or NrdI). Likewise, an NrdG candidate is only accepted if located close to NrdD, or when this criterion is not valid only the highest scoring NrdG candidate is accepted for an organism with an existing NrdD entry.

The alignment of candidates to known experimentally annotated RNR sequences also provides an initial indication of potential presence of self-splicing introns and inteins in the RNRs. Putative intein sequences within candidate RNR sequences are manually curated with the aid of the BLAST function of the InBase database (The Intein Database and Registry, http://www.neb.com/neb/inteins.html) [21]. Candidate selfsplicing intron sequences are identified within RNR genes by manual secondary structure folding of the presumed intronic RNA according to the conventional folding suggested by Cech et al. [22].

Instead of using a release scheme for database content, the database is continuously updated with new sequences. In contrast, the database user interface is under a release scheme, and is currently at version 1.3. On each page, the date when data was last inserted or corrected is displayed together with the version number of the interface. At the time of writing (July 2009), the database contains over 2000 cellular organisms and viruses and over 9000 protein sequences (Table 2). The main sequence data source is GenBank, but this is augmented at times with other data sources when quality sequences are available that have not yet been uploaded to GenBank. At the time of writing, we have downloaded and screened additional sequence data from the Joint Genome Institute http://www.jgi.doe.gov/, the Broad Institute http://www.broad.mit.edu/ and the University of Tokyo Cyanidioschyzon merolae Genome Project database http://merolae.biol.s.u-tokyo.ac.jp/ in addition to data from GenBank.

Structures for representatives for all RNR proteins except the class III activase, NrdG, and the regulatory protein NrdR, have been solved. RNRdb contains annotations and descriptions for all published RNR structures, together with links to the structure files in Protein Data Bank http://www.rcsb.org/pdb/.

Each RNRdb entry contains the full amino acid sequence and cross-references to the source databases for sequence and protein structure information, as well as genomic location, when known. In addition to the classification of each sequence (by class and subunit), additional attributes are listed, which enables retrieval of proteins with solved structures, experimentally derived mutational data (including the corresponding PubMed references), and presence of intervening self-splicing sequences, i.e. group I and II introns and inteins; these are cross referenced when applicable. The system for managing attributes is flexibly implemented, allowing new classification attributes to be added during curation.

Each sequence is linked to a source organism or virus record, which in turn is linked to its full NCBI taxonomy hierarchy allowing filtering of sequences based on taxonomy (see below). Organisms and viruses with fully sequenced genomes are labelled, making it possible to establish whether, for any given organism, the list of annotated RNRs is based on complete or incomplete genome sequence data. RNRdb also contains information about genomes that lack RNRs (determined through candidate screening of complete genome sequences, as described above). As of July 2009 there are only five such cases among cellular organisms, three bacteria (*Borrelia burgdorferi *[23,24], *Buchnera aphidicola *str. Cc [25] and *Ureaplasma urealyticum *[26,27]) and two eukaryotes (*Entamoeba histolytica *[28,29] and *Giardia lamblia *[30,31]). These are all parasites or obligate intracellular endosymbionts, and absence of RNRs indicates that all must rely on salvage of hostderived deoxyribonucleotides.

## Utility of RNRdb

### Motivation for building a specialist RNR database

There are numerous large-scale sequence databases that provide annotations and classifications of proteins and protein families. In some cases, the process of annotation is approached systematically [32-34] while in others, e.g. GenBank, annotations are made by those depositing information, using a variety of methodologies. Specialist databases, in contrast, can serve to collate all available information on a given protein or protein family, and are small enough to allow detailed, expert curation. It is for these reasons that we were originally motivated to build RNRdb. That said, there is only any real value in such an endeavour if expert curation results in significant improvements in accuracy over existing resources. To this end, we compared our annotations to those recorded in GenBank entries for all sequences in RNRdb on the basis of the following criteria: RNR class (I, II or III), protein role (subunit identity -- as detailed in Table 1, with the additional inclusion of NrdG, NrdH, NrdI & NrdR proteins), general function (i.e. involved in ribonucleotide metabolism) and identification of the ATP cone [35], where present. We found that GenBank annotations are surprisingly inaccurate (Table 3). Only 23% of entries in RNRdb were correctly annotated by class, role and function, and 17% of sequences included in RNRdb on the basis of manual curation are incorrectly annotated for all criteria. In addition, over 100 group I introns and ca 80 inteins have been manually curated from the entries. This serves to highlight the potential value of manual curation, given the complexities of annotation [20,36]. While manual curation is not infallible (see previous section), we nevertheless believe that our annotations are significantly improved over existing publicly available annotations.

**Table 3:** RNR annotation

Correct class	Correct role	Correct function	Single domain	N	Percent
Yes	Yes	Yes	NA	1702	23

Yes	No	Yes	NA	569	8

No	Yes	NA	NA	2339	32

No	No	Yes	NA	1185	16

No	No	Almost^a^	NA	196	3

No	No	No	Yes	105	1

No	No	No	No	1155	16

**Sums**				7251	100

GenBank definitions of proteins in RNRdb. Four different aspects of RNR functional annotation have been investigated: RNR class, protein role (subunit identity), general function (involved with ribonucleotides) and single domain identification (the ATP cone [35] that regulates activity in many enzymatic subunits). Only NCBI entries for full-length sequences were used, and nine sequences that were difficult to classify were excluded. NA, not applicable.

^a ^Proteins annotated such that they contain the string "ribonucl" for all RNR proteins, or "repressor" for NrdR, but not a full description of the function such that they would classify as correctly functionally annotated, have been classified as almost correctly annotated with respect to function.

## Querying RNRdb

The RNRdb homepage contains tabs to a short introduction on RNRs ("About RNRs"), a glossary, and a list of key literature references ("Bibliography"). We have focused the user interface of the database on tools for exploration of RNR diversity and distribution and to serve as an annotation resource for specialists. There are four main access points to RNR sequences in the database:

i) The "RNRs by organism" page presents the entire database in tabular format. By scrolling down the page or using the browser to search for text strings, the user can explore the distribution of RNRs in the three cellular domains as well as among viruses. Proteins with additional attributes (solved structure, mutagenised forms, selfsplicing introns and inteins) and fully sequenced genomes are indicated by red superscript abbreviations; clicking on these superscripts links to explanations. Following an organism or protein hyperlink presents the user with all RNR sequences for that organism or the chosen protein respectively, together with classification information and cross-references to other databases.

ii) The "Search" page enables searches of the database at any taxonomic level, ranging from all cellular domains and viruses down to single species. Furthermore, organisms possessing or lacking particular RNR classes and/or the NrdR regulator can be retrieved. It is also possible to retrieve proteins with specific annotated attributes (e.g. inteins) or to restrict the search to completely sequenced genomes. All three aspects of searches can also be combined, allowing searches for, e.g., enterobacterial class I RNRs for which solved structures exist.

iii) The "BLAST" [37] page permits searches of RNRdb using either protein (blastp) or DNA sequence (blastx) data. The BLAST search interface can be used to annotate unknown sequences, or to investigate annotations in other databases.

iv) The "Statistics" page provides tabulated summaries of RNR distribution across the three cellular domains, and DNA viruses, both for complete genomes and for the entire database (Table 2 and 4). For complete genomes, all combinations of RNR classes are summed for each domain (likewise viruses) and across domains; clicking on sums generates a detailed list of species and proteins (e.g. archaea carrying class II and class III RNRs).

**Table 4:** RNR distribution

Combination	Archaea	Archaeal viruses	Bacteria	Bacteriophages	Eukaryotes	Eukaryotic viruses	Totals
I	0	0	211	18	56	81	366

II	20	1	87	14	1	0	123

III	15	0	11	1	0	0	27

I+II	2	0	82	0	2	1	87

I+III	0	0	222	14	1	0	237

II+III	15	0	47	0	0	0	62

I+II+III	1	0	54	0	2	0	57

None	0	0	3	0	2	0	5

Totals	53	1	717	47	64	82	964

Combinations of RNR classes in the cellular domains and viruses. This table only contains data from fully sequenced genomes. We have exhaustively verified absence of RNRs in organismal, but not in viral genomes.

To facilitate data acquisition for comparative analyses, sequences (including those returned by a specific search) can be downloaded in FASTA or NEXUS format via the protein detail pages. Subsets of sequence data from returned searches or from the "RNRs by organism" page can also be selected manually via checkboxes and downloaded as above.

## Discussion

Our knowledge of the distribution of ribonucleotide reductases has expanded rapidly over the last few years. Until recently, the distribution of the three classes was considered rather limited, and, as a domain, only bacteria were thought to possess the full gamut of classes. Class I RNRs were thought to be absent from archaea, and no sequences for classes II and III were known from eukaryotes. While whole genome data have expanded this picture, annotations in other public databases (mainly GenBank) are often uninformative as regards RNR class and subunit type (meaning that this has to be checked manually) (Table 3). Moreover, a number of genomes carry clear misannotations, and protein family databases do not always correctly categorise RNRs. Searching Pfam [32], for instance, returns adequate descriptions of two of the structural domains ("ATP cone" and "Glycine radical") of the *Escherichia coli *K12 catalytic subunit class III protein, but no family or structural domain with clear reference to RNRs. Searching with the *E. coli *K12 class Ia catalytic subunit protein sequence and the *Thermotoga maritima *class II protein sequence returns in both cases the "Ribonucleotide reductase, all-alpha domain" and the "Ribonucleotide reductase, barrel domain" family. It is thus difficult to tell from Pfam searches that the class III sequence is an RNR and that the class I and class II sequences are from different classes. Although other protein family databases have broader coverage (e.g. Pfam [32], InterPro [33] and PhyloFacts [34]), our approach with HMM profiles followed by manual curation yields more accurate descriptions (unpublished observations).

RNRdb thus offers a first clear overview of the distribution of the three classes of ribonucleotide reductase. The data curated in RNRdb make it clear that all three classes of ribonucleotide reductase are found in all three organismal domains. Around half of all sequenced species carry genes for only one class of RNR, but among those with more than one RNR class, two eukaryotes, one archeon, and 54 (7.5%) of the fully sequenced bacterial genomes harbour genes for all three RNR classes (Table 4). The varying environmental and biochemical conditions under which each class of RNR can synthesise deoxyribonucleotides (Table 1), the complex distribution of the three classes across genomes, and the frequent presence of more than one complete set of RNR genes per genome suggests a role for horizontal gene transfer in forming this distribution. Evolutionary genomic analyses support this view (Lundin et al., in prep.).

## Conclusion and future directions

The diverse distribution of ribonucleotide reductases was poorly appreciated prior to the genomic era in biological research. Prior to the establishment of RNRdb this information was difficult to navigate due to incomplete and misleading annotation regarding class membership and subcomponent information in databases. We demonstrate that manual curation of protein sequences leads to significant improvements over existing annotations, and that there is therefore value in generating such annotation sets. Indeed, there are ongoing efforts to try and integrate such approaches to large-scale annotation [38].

Our plans for the next major release of the database, RNRdb 2.0, include tools to enable users to explore sequence diversity within the components of different RNR classes. Specifically, we are developing tools to complement the current BLAST search feature with a service that matches user submitted sequences to our set of HMM profiles, allowing a more precise and fine-grained annotation.

Interestingly, ribonucleotide reductases are the most abundant enzyme family identified in metagenomic sequencing projects [39], and the potential utility of relating the biochemical attributes of RNRs to environmental parameters such as oxygen levels or iron availability is clear. RNRdb 2.0 will therefore also include sequences from environmental samples and other sources where the identity of the organism cannot be established. Our vision for RNRdb 2.0 is a database where the user can explore sequence space to analyse not only which classes exist in different taxa, but also in which organisms and environments subtypes of RNR genes occur. We will continue to expand the content and scope of RNRdb, in order to further deepen our understanding of this fascinating enzyme, and to explore its utility in the metagenomic analyses of diverse microbial environments.

## Availability and requirements

RNRdb is freely available at http://rnrdb.molbio.su.se.

## Authors' contributions

BMS collated the initial dataset. DL designed and programmed the database and candidate search programs. BMS and AMP contributed to the design phase. BMS and ET curated the data. DL, AMP and BMS wrote the manuscript. All authors read and approved the final version.
